# The identification of job opportunities for severely disabled sick-listed employees

**DOI:** 10.1186/1471-2458-12-156

**Published:** 2012-03-06

**Authors:** Jake PJ Broersen, Henny PG Mulders, Antonius JM Schellart, Allard J van der Beek

**Affiliations:** 1 Department of Public and Occupational Health, EMGO Institute for Health and Care Research, VU University Medical Centre, Van der Boechorststraat 7, NL-1081 BT Amsterdam, The Netherlands; 2 Research Centre for Insurance Medicine, collaboration between AMC-UMCG-UWV-VUmc, Van der Boechorststraat 7, NL-1081 BT Amsterdam, The Netherlands; 3 Knowledge Centre of the Employee Insurance Authority, La Guardiaweg 36-162, NL-1043 DJ Amsterdam, The Netherlands

## Abstract

**Background:**

Work disability is a major problem for both the worker and society. To explore the work opportunities in regular jobs of persons low in functional abilities, we tried to identify occupations low in task demands. Because of the variety of functional abilities and of the corresponding work demands, the disabled persons need to be classified by type of disability in a limited number of subgroups. Within each subgroup, occupations judged suitable for the most seriously disabled will be selected as having a very low level of the corresponding task demands. These occupations can be applied as reference occupations to assess the presence or absence of work capacity of sick-listed employees in regular jobs, and as job opportunities for people with a specific type of functional disability.

**Methods:**

Registered data from 50,931 disability assessments within the Dutch social security system were used in a second order factor analysis to identify types of disabilities in claimants for a disability pension. Threshold values were chosen to classify claimants according to the severity of the disability. In the disability assessment procedure, a labour expert needs to select jobs with task demands not exceeding the functional abilities of the claimant. For each type of disability, the accessible jobs for the subgroup of the most severely disabled claimants were identified as lowest in the corresponding demand.

**Results:**

The factor analysis resulted in four types of disabilities: general physical ability; autonomy; psychological ability; and manual skills. For each of these types of disablement, a set of four to six occupations low in task demands were selected for the subgroup of most severely disabled claimants. Because of an overlap of the sets of occupations, 13 occupations were selected in total. The percentage of claimants with at least one of the occupations of the corresponding set (the coverage), ranged from 84% to 93%. An alternative selection of six occupations for all subgroups with even less overlap had a coverage ranging from 84% to 89% per subgroup.

**Conclusion:**

This study resulted in two proposals for a set of reference occupations. Further research will be needed to compare the results of the new method of disability assessment to the results of the method presently used in practice.

## Background

Poor health resulting in long-term sickness absence and work disability is a major problem for the employee involved as well as for the larger society. The Organisation for Economic Co-operation and Development (OECD) studied the extent of this problem of long-term sickness absence and work disability, using data from some of the member states. The costs of sickness absence and work disability in those countries were estimated to be 1.9% of the Gross Domestic Product in 2007 [1]. A study within the European Union estimated the prevalence rate of long-standing health problems or disability in the working-age population to be 15.7% [2,3].

A mismatch of the work capacity of an employee with a health problem on the one hand and the task demands in his/her job on the other may lead to sickness absence. Such a mismatch is a consequence of impairments and disabilities caused by the health problem and a lack of possibilities to adjust the job demands to the diminished work capacity of the employee. The term 'impairment' is used to indicate a medical condition that leads to disability, and 'disability' refers to a restriction in functioning or an activity limitation. Mostly, the afore-mentioned mismatch will be temporary, and the employee will resume work after recovery from a sickness spell. In some cases, an employee does not (fully) recover over time, and will remain disabled in his/her work to some extent. If this disability is only minor, the employee may be able to resume his/her own work with increased effort, eventually with minor adaptations of the task demands, tailored to his/her disability. In case the disability is more severe, major interventions in the person domain and/or in the job will be required to facilitate return to work. Examples of such interventions are: enhancement of abilities by education and by training of skills; adaptation of the task demands, and so on [4-6]. Another way to deal with the consequences of the medical situation of these employees may be to reappoint him/her to another job, more suited to his or her reduced abilities.

If the work ability of an employee is affected by health problems in such severe way that return to his/her former job is impossible, then the reduced work ability of the employee will impose limitations on the search for job alternatives. The accessibility of a job for an employee with a specific type of disability will partly be dependent of the (maximum) level of the corresponding task demands in that job. As those demands in the job increase, jobs will be accessible to a lower percentage of sick-listed employees with that disability. Besides, as the severity of the disability increases, the dependency on low-demand jobs will increase, and the number of accessible regular job alternatives will decrease (in this manuscript, the expression 'low-demand jobs' is used to indicate jobs low in task demands). The plausibility of such a negative relationship can be illustrated with the results from a literature review by Turner et al. [7], who reported positive relations between (self-reported) pain and functional disability on the one hand and subsequent duration of work disability on the other.

**Figure 1:**
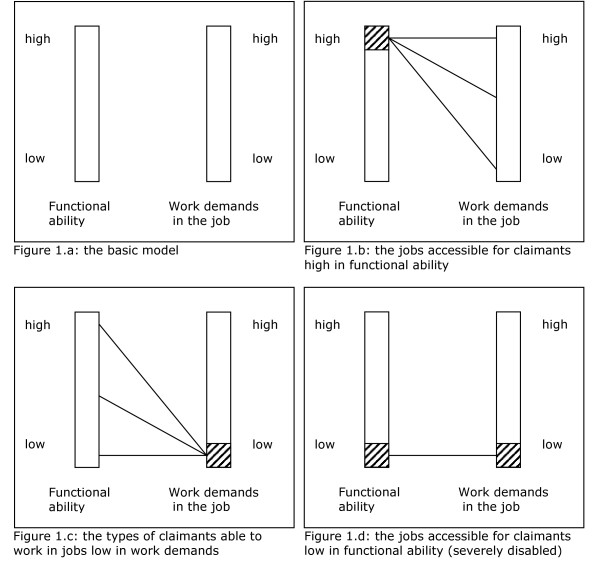
**A simple model of work load and work capacity, with only one dimension for the work demands in the job and for the functional abilities of the claimant**.

We can illustrate the relation between the functional abilities of an employee and the task demands in a job with a simple, one-dimensional model. In this simple model, the work load of a job is a one-dimensional work demand, measured by a single parameter, and the corresponding one-dimensional functional ability determines whether an employee is able to cope with the work demand (Figure 1a). Our model excludes jobs with major adaptations - for example in sheltered workshops - and employees too severely disabled to work in any regular job. The functional ability of employees increases from low to high, and employees can be sorted accordingly. Likewise, jobs can be sorted on work demand from low to high. Employees high on functional ability have basically access to all jobs (Figure 1b). Conversely, jobs very low in work demand are in principle accessible to all employees who can work in a regular job (Figure 1c). For employees low on functional abilities (i.e. relatively severely disabled, but still able to work), the number of accessible jobs is restricted: they can only work in jobs with the lowest level of work demand (Figure 1d). By selecting employees low on functional abilities and studying the regular jobs accessible to them, one can also identify jobs lowest in task demand, which are the few accessible jobs for these employees. In addition, these low-demand jobs can be used as a classification instrument, to distinguish two groups. The first group consists of all employees whose functional abilities minimally equal the work demands in these low-demand jobs: they have a remaining capacity to work in normal jobs. This group includes all employees who still are able to work in regular jobs (Figure 1c). The second group consists of the employees for whom the work demands in the afore-mentioned low-demand jobs exceed their functional abilities. In our simple model, the work demands in all jobs exceed those in the low-demand jobs, and therefore the employees in the second group are not able to work in any regular job.

In reality, the functional disability of long-term sick-listed employees varies not only in severity, but also in nature. Moreover, combinations of health problems within one employee will occur (co-morbidity), mostly resulting in combinations of functional disabilities. Within one specific type of disability, the severity of the disability of an employee will determine the number of accessible jobs, and the most severely disabled are not able to work at all in regular jobs, i.e. in jobs without major adaptations, just as in the afore-mentioned simple model. Employees whose work disability is a little bit less severe will be on the borderline between being able to work and work disability. We expect that only a restricted number of jobs will be suitable/accessible for them, just as in the simple model (Figure 1d). For employees having a specific type of disability of a 'medium' severity, jobs low on the corresponding demands are the rare opportunities for them to work in a regular job. The composition of a set of low-demand jobs should be comprehensive to contain accessible jobs for as many disabled employees as possible on the one hand, while the size of the set of jobs has to be manageable on the other hand.

The size of a set of jobs can be reduced by classifying those jobs according to their occupational title into occupations, for example according to the Standard Classification of Occupations (abbr. SCO-'92) of Statistics Netherlands CBS [8]. The SCO-92 distinguishes 1211 occupations to classify all jobs in the Netherlands. Therefore, there is a considerable diversity in task demands and working conditions between occupations. Given a specific type of disability, occupations very low in the corresponding demands can be identified. In rehabilitation efforts for disabled employees with that type of disablement, the low-demand occupations have to be considered for job opportunities. Moreover, if an employee with this specific type of disability cannot work in any of the corresponding low-demand occupations, he/she will not be able to work in any regular occupation, and major efforts will be needed in job adjustments; or work in supported employment may be necessary; or a work disability pension remains as a last resort. This way, the presence or absence of work capacity for regular jobs for employees with the specific type of disability can be assessed by using those low-demand occupations: the so-called reference occupations.

Regular occupations still accessible for severely disabled employees are the most eligible to be used as reference occupations. To identify those reference occupations, firstly functional abilities of sick-listed employees have to be summarised in a limited number of scale variables, each of which represents a common type of disability of the claimants. Secondly, subgroups of claimants with the same main type of disability need to be identified, characterised by high scores on one of the scale variables, and relatively low scores on the other scale variables. Thirdly, the jobs judged to be accessible to such a subgroup by a labour expert in work disability assessments need to be classified into occupations according to the SCO-'92 [8]. Fourthly, the occupations most frequently used need to be presented as potential reference occupations.

The indirect method of selecting low-demand jobs, using the data of severely disabled employees and the jobs accessible to them, seems a cumbersome manner to identify those jobs. It may seem more effective to measure specific task demands in a variety of jobs, and to select the jobs lowest in those demands [9,10]. However, the low level of some of the task demands may be combined by high levels of other demands, or with other thresholds, for example the required education. A labour expert takes all these factors into account in selecting suitable jobs for claimants in the work disability assessment procedure, which justifies this indirect method of selecting low-demand jobs.

In Norway, data about functional capacities of sick-listed employees were summarised in seven scales, based on analyses resembling the analyses for the first step in our identification process of reference occupations, as proposed above. The functional capacities of sick-listed employees in Norway have been registered using the Norwegian Function Assessment Scale (NFAS) [11,12], consisting of 40 items. Four scales about physical dimensions of functioning were identified, i.e. walking/standing; holding/handling; lifting/carrying; and sitting. The other three scales measured mental dimensions: coping; communication; and the senses. The seven scales of the NFAS were developed to be used by rehabilitation professionals to assess the work capacity of sick-listed employees.

To reach the objective of our study, the first step is to identify a limited number of types of functional disabilities in long-term sick-listed employees, to create groups relatively homogeneous in the nature of their disabilities. Subsequently, the subgroup of employees most severely disabled within each type of disability will be selected, as well as the jobs still accessible to them. Those jobs, low in the corresponding task demands, can be aggregated into occupations and the occupations most frequently used can be applied as reference occupations to assess the presence or absence of work capacity of sick-listed employees in regular jobs, and as job opportunities for people with a specific type of functional disability. The application of the reference occupations will have to be studied in future research.

## Methods

This study was based on secondary analyses of Dutch social security data. In the social security system of the Netherlands, the work capacity of each sick-listed employee is assessed after two years of sickness absence. In the assessment procedure, an Insurance physician (IP) assesses a broad variety of functional abilities, and registers the results in a standardized List of Functional Abilities (LFA) [13]. The LFA was partly based on the International Classification of Functioning, Disability and Health (ICF) [14]. In a next step, a labour expert (LE) matches the functional abilities of the employee to the work demands of a set of 7,000 heterogeneous regular jobs, using the computer system CAMS: the Claim Assessment and Monitoring System [13,15]. The task demands of the jobs in the job file of the CAMS were based on on-site observations by specialised LEs, with scheduled updates. The jobs in the job file of the CAMS were classified into occupations according to the SCO-'92 of Statistics Netherlands CBS [8]. This classification was largely based on the International Standard Classification of Occupations (ISCO-88) of the International Labour Organisation [16], and comprises 1,211 distinct occupations. The primary criterion of the SCO-'92 is the level of the educational requirements of the occupation, with 5 classes of increasing level: elementary; low; medium; high; and academic [8]. The number of occupational titles per level in the SCO-'92 is presented in the first row of Table 1.

**Table 1:** Number of occupations within occupational level of the SBC-'92, and the number of occupations used in the Claim Assessment and Monitoring System (CAMS)

Number of occupations within occupational level of the SBC-'92, and the number used in the CAMS						
			Occupational level			
	elementary	low	medium	high	academic	Total
Total number of occupations within the SBC-'92	34	202	396	339	240	1211
Number of occupations in the CAMS (percentage of the total number of SBC-'92 occupations)	16 (47%)	79 (39%)	92 (23%)	47 (14%)	8 (3%)	244 (20%)

The LFA and some other employee characteristics, and the jobs selected in the assessment process as accessible to him/her, were registered/monitored by the computer system CAMS. The cumulative files containing the LFA data of the claimants were previously used to investigate the dimensional structure within the items of the LFA [17], and this resulted in 15 sum variables, to be used in various future applications. However, the number of 15 variables was too large for our present purpose to identify a limited number of dimensions within functional disabilities, and to reduce that number we used a second order factor analysis. The Cronbach's Alpha of one of the 15 sum variables ('Communication') was only 0.54 [17], and therefore we excluded this variable from the present analysis. The second order factors, based on the LFA data of 50,931 claimants, were used to aggregate the items into new scales.

The interrelations between the first order factors were analyzed using factor analysis of the SPSS package, with principal components analysis and varimax rotation. Furthermore, reliability analyses (Cronbach's Alpha) of the scales were conducted.

Within each of the new scale variables, the relation between the value of the sum variable, irrespective of the value of the other sum variables, and the probability of entitlement to a full disability pension was used to classify the values as low, medium or high. For this purpose, two threshold values were set, arbitrarily, at 50% and 66% probability. For each of the sum variables, the monotonic relation between the scale variable and the probability of total disability entitlement were used to identify the two threshold values for 50% and 66% probability. For each sum variable, a group of claimants was selected with high scores on that sum variable (> 66% probability) and low scores on the other sum variables (< 50% probability). Therefore, each group had one main disablement. To be used in the selection of reference occupations, the claimants had to be assessed as able to work in regular jobs. This outcome of the assessment was based on the identification of a minimum of three suitable occupations from the job file of the CAMS, each occupation with at least three observations of actually existing jobs [13]. The occupation most frequently selected for a subgroup of severely disabled claimants was the most eligible candidate to be selected as reference occupation.

Within each of the distinguished subgroups of severely disabled employees, the use of each occupation in the disability assessment procedures was computed as percentage of the total number in the subgroup. The occupation with the highest percentage was the most eligible candidate as a reference occupation. If this most frequently selected occupation was not used in the assessments of all clients, then other occupations had been used for some of the severely disabled clients. The same procedure was repeated within this group of claimants, and the most frequently selected occupation for this group was added to the first to create a set of reference occupations with maximum total cumulative coverage. This addition of occupations was repeated until the additional coverage was almost asymptotic, i.e. only a few extra employees were covered. To keep the assessment method manageable, the total number of occupations within type of disability had to be limited, and therefore the number of occupations was considered as an extra criterion in the ultimate choice of a set of reference occupations.

## Results

The second order factor analyses on the 14 sum variables resulted in four second order factors. The factor loadings are presented in Table 2. The title/common subject of the four second order factors were: general physical ability, covering various aspects of the musculoskeletal system; autonomy, i.e. to act autonomously and independently in the working situation; the abilities to cope with various psychological task demands; and manual skills and grip strength. The Cronbach's Alphas were 0.68, 0.63, 0.66, and 0.46, respectively.

**Table 2:** The factor loadings of 14 sum variables on four second order factors

Rotated Component Matrix				
	**Factor (a)**			

	**1**	**2**	**3**	**4**

The use of the legs	.815			
The use of the arms	.806			.332
The movements of the trunk/back	.804			
The posture of the trunk/back	.742			
The use of the neck	.319			
No independence in performance		.801		
Acting efficiently		.782		
Taking initiative		.688		
Work stress			.837	
Social task demands		.318	.708	
Cognitive functioning		.463	.660	
Working hours			.623	
Grip of the hands				.831
The use of the hands and fingers	.316			.808

Extraction Method: Principal Component Analysis

Rotation Method: Varimax with Kaiser Normalization

(a) Second order factors:

1: General physical abilities

2: Autonomous and independent task performance

3: Psychological abilities

4: Manual skills and grip strength

Four new sum variables were based on these analyses, and the relation between the sum score and the risk of an assessment result of total work disablement was established. Four groups of claimants with one main type of disability were selected, each with a score above the high threshold value on one sum variable and scores below the low threshold value on the three other sum variables. According to the LE, some claimants within each of those groups were able to work in normal jobs, notwithstanding their high sum score on one of the LFA-scales. Those normal jobs were classified into low-demand occupations for that type of disability. Table 3 lists some of the characteristics of those four subgroups of claimants, and of the group of all other claimants not belonging to the former four subgroups.

**Table 3:** Characteristics of four subgroups of severely disabled claimants and of the other claimants

	Subgroup of claimants (a)				
**Characteristics of the claimant**	**Physical**	**Auton**.	**Psych**.	**Hand**	**Other**

Sex:					
Male	38%	53%	36%	49%	47%
Female	62%	47%	64%	51%	53%
Age group:					
< 25 years	2%	3%	4%	3%	2%
25 - 35 years	11%	28%	22%	17%	16%
35 - 45 years	24%	30%	33%	30%	26%
45 - 55 years	34%	25%	28%	29%	32%
> = 55 years	29%	14%	13%	22%	24%
Educational level:					
Primary education	25%	40%	29%	29%	31%
Lower secundary education	35%	16%	31%	33%	33%
Medium secundary education	31%	27%	30%	32%	27%
Higher or university education	10%	17%	11%	5%	10%
Total number of claimants	**371**	**118**	**335**	**230**	**49,877**

(a) Subgroup of claimants, disabled in:

Physical: general physical abilities

Auton.: autonomous and independent task performance

Psych.: psychological abilities

Hand: manual skills and grip strength

Other: All claimants not belonging to the four above-mentioned subgroups

The most accessible occupations of the four subgroups of severely disabled of claimants are presented in Table 4. The educational level of all of these jobs was 'elementary' or 'low'. Within each of the four subgroups of employees, four to six occupations were sufficient to cover about 90% of the assessments. For the claimants in subgroups 1 and 4, i.e. disabled in general physical ability and manual skills, respectively, the occupational level of most of the suitable jobs was 'low'. Five out of the six most suitable jobs for subgroup 3 with psychological disabilities were on the elementary level, and for the claimants of subgroup 2 (autonomy) two occupations of each level were most frequently used.

**Table 4:** The occupations most frequent used in work disability assessments of four subgroups of severely disabled claimants, and percentages of claimants for whom at least one of the occupations was used

	Subgroup of claimants (a)			
**Occupation**	**Physical**	**Auton**.	**Psych**.	**Hand**

Typist/telephonist/receptionist	x (b)			x
Operator sewing machine (in industry)	x	x	x	
Receptionist, desk clerk	x			
Administrative employee (minor level)	x			
Assembler electrical devices	x	x		
Delivery man/driver delivery van	x			x
Car-park attendant				x
Security man/surveillant				x
Employee manufacturing (manual)		x	x	
Employee manual packing		x	x	
Employee domestic services			x	
Cleaner			x	
Assistant agricultural worker			x	
Number of occupations in the selection	6	4	6	4
Percentage of claimants for whom one or more of the (four or six) selected occupations was used in the disability assessment	92%	84%	93%	89%
Percentage of claimants for whom one or more of all thirteen occupations was used in the disability assessment	95%	94%	96%	94%

(a) Subgroup of claimants, disabled in:

Physical: general physical abilities

Auton.: autonomous and independent task performance

Psych.: psychological abilities

Hand: manual skills and grip strength

(b) In the row behind the title of the occupation, the column of the subgroup of claimants is marked with an "x" if the occupation belonged to the selection of most frequently used occupations for that subgroup of claimants

For many of the assessments of the four subgroups of claimants, more than one of the selected four or six occupations were used. Moreover, some occupations appeared in the list of the most frequently selected occupations of more than one subgroup of claimants. Five occupations were frequently used for two subgroups of claimants, and one occupation for three subgroups (Table 4). By selecting a subset of six out of the 13 occupations of Table 4, we took advantage of the overlap in frequently selected occupations between subgroups of claimants, and so we reduced the redundancy. In this way, we were able to explore the coverage of such a limited number of occupations over all subgroups of claimants. The choice of occupations was based on both the accessibility within each subgroup of claimants and the overlap in accessible occupations between subgroups of claimants. The coverage of the four subgroups of claimants with the six selected occupations are presented in Table 5. The coverage per subgroup of claimants varied from 84% to 89%. The weighted mean coverage for all four selected subgroups of claimants was almost 87% vs. almost 91% for the four separate sets of Table 4. The coverage of the six general occupations of Table 5 roughly equalled the coverage of the four specific occupations of Table 4 for the second and fourth subgroup of claimants. The reduction in coverage, compared to the four specific sets of occupations per subgroup of claimants, was relatively large in the third subgroup. However, the overall mean coverage per occupation increased considerable because of the decrease in the number of occupations: from 13 to six occupations. Depending on the kind of application, one can choose between the coverage of a large majority of claimants by a restricted set of six occupations of Table 5 and a more comprehensive coverage by the broader set of 13 occupations of Table 4.

**Table 5:** A selection of six reference occupations, and the number (and percentage) of assessments of four subgroups of claimants these jobs were used in

	Subgroup of claimants (a)			
Occupation	Physical	Auton.	Psych.	Hand
Typist/telephonist/receptionist	184	10	21	78
Operator sewing machine (in industry)	128	41	177	22
Delivery man/driver delivery van	81	5	19	70
Car-park attendant	34	3	0	149
Employee manufacturing (manual)	10	60	170	4
Employee manual packing	66	50	142	9
Number of assessments in which at least one of the six occupations was used	326	99	282	205
Total number of assessments within subgroup of claimants	371	118	335	230
Percentage of the total number assessments within subgroup of claimants in which at least one of the six occupations was used	88%	84%	84%	89%

(a) Subgroup of claimants, disabled in:

Physical: general physical abilities

Auton.: autonomous and independent task performance

Psych.: psychological abilities

Hand: manual skills and grip strength

## Discussion

The analysis of the interrelations between functional disabilities of claimants for a disability pension produced four main types of disabilities. On the basis of those four types of disabilities, we identified four subgroups of severely disabled claimants who were still able to work in normal jobs, and were relatively homogeneous in the main type of disablement. Within each of those subgroups, jobs that were regarded as accessible for them were classified into occupations. The occupations most frequently used for the claimants within each of the four subgroups were selected to become reference occupations. The reference occupations can be used to direct the rehabilitation efforts towards accessible work opportunities, and may be applicable in the assessment of the work capacity of claimants in regular jobs. In the search for a job for a claimant with a severe disability of one of the four types, and restricting the search to regular jobs without major adaptations, one should start with considering the reference occupations belonging to that type of disability. If the claimant is able to work in one of the reference occupations, one can subsequently look for further job opportunities, possibly with higher task demands, also depending of the characteristics of the client, e.g. education; work experience; motivation; and so on. On the other hand, if a claimant is not able to work in any of the reference occupations, it is unlikely that any other occupation is accessible for that claimant because of the severity of his/her disability and the higher task demands in those other regular occupations. Therefore, the reference occupations may be useful instruments to assess the possibilities of claimants to work in regular jobs at all, i.e. in jobs without major adjustments. The reference occupations were selected because their frequent use in disability assessments for one of the four subgroups, and therefore the corresponding work demands are very low. Especially for the severely disabled these occupations are the rare opportunities to work in regular jobs. Moreover, none of the work demands in these occupations reaches a level that forms a threshold for many claimants, so the general accessibility of these occupations is relatively high, also for people with other disabilities.

Four scale variables were computed to measure the four disability types. The Cronbach's Alpha of one of those scales was only 0.46, probably due to the low number of two variables on which the scale was based. For the application of the scale scores in the identification of subgroups of severely disabled claimants in this study, the low value of the Cronbach's Alpha of this scale was considered acceptable.

To identify severely disabled claimants with one of the four types of disabilities (but still assessed as being able to work), two threshold values were arbitrarily set at 50% and 66% probability of a full disability pension. Additional sensitivity analyses in the vicinity of the 66% threshold did not show a sudden change in accessible occupations (results not presented here), indicating that the choice of the threshold level did not dominate the selection of reference occupations.

The four subgroups of severely disabled claimants differed in some characteristics from each other and from the other claimants (Table 3). The subgroups disabled in psychological abilities and autonomy were more represented in the two younger age categories, especially compared to the subgroup disabled in general physical abilities. In the two subgroups disabled in general physical abilities and in psychological abilities included a higher percentage of female claimants. The four disabled subgroups were better represented in two higher educational categories taken together than the group of all other claimants. However, the results for the four educational categories separately differed between the four subgroups severely disabled claimants. Although some of the differences between the groups in the three characteristics of Table 3 were substantial, none of these were indications that the four subgroups of severely disabled claimants were an extremely deviating selection from the total group of claimants.

The second order factor analysis on 14 of the 15 first order factors resulted in four factors, which was less than the seven factors that were identified within the Norwegian NFAS [11,12]. The Norwegian scale "holding/handling" showed a great resemblance to the scale "manual skill and grip strength". Within the NFAS, three other scales within the physical domain were distinguished, whereas in our second order factor analysis the remaining factors were combined to produce the scale "general physical abilities". Of the three factors in the mental domain of the NFAS, the scale "senses" resembled the scale "communication" of the first order factor analysis of the LFA, which we excluded from our analysis because of the lack of reliability. The remaining two scales in the mental domain of the NFAS differed from the two psycho-social scales of the LFA. This may be due to differences in the subject matter and the application between the two functional ability lists, although both lists were partly based on the ICF [14]. The main differences were: an emphasis on everyday activities vs. work-related activities; self-reported disability vs. assessment by an insurance physician; and duration of the sickness spell preceding the disability assessment: six weeks vs. two years.

The similarities and differences between the factors found in our study and in the Norwegian study [11,12] suggest limited possibilities to apply our results in other countries. It seems plausible that the relative prevalence of some types of disabilities in the work force will show similarities between countries, especially under similar conditions with respect to, for example, the level of prosperity; the nature of the jobs; the working population; the social security system; and so on. Rehabilitation professionals in other countries, consulted by disabled clients of such a similar type, may be inspired by our reference occupations in their advising about accessible occupations, although the task composition of specific jobs may differ between countries. In addition, the method for the selection of reference occupations may be applied in other countries, although the availability of a similar data set may be problem.

In the work disability assessment procedure, a job file with a total set of about 7,000 jobs was used to select suitable jobs from. Although this number of jobs was substantial, these jobs constituted only a limited part of the total of millions of regular jobs in the Netherlands. The size of the job file of the CAMS was limited because of the costs of the observations on location of the task demands of jobs within companies and of the scheduled updates of those observations. The coverage of the total of 1,211 occupational titles of the SCO-'92 by the occupations in the CAMS was relatively high in the three lower level occupations (coverage 47%; 39%; and 23%, respectively), whereas the occupations on the high level (14%) and the academic level (3%) were relatively sparsely covered (the second row of Table 1). In the development and maintenance of the CAMS job file, this unbalanced composition of the job file was preferred because the lower level jobs were accessible to almost all claimants as far as the required educational level is concerned, while higher level jobs were only accessible to the higher educated claimants with the right specialization. However, the selection of only elementary and low level occupations as reference occupations was not only caused by the composition of the CAMS job file. Although the education requirements of high and academic level occupations that were included in the job file were all of a more or less general nature to avoid additional barriers caused by the required specialization, those educational requirements would only be met by a small part of the work force (and of the claimants). Some task demands in a number of higher-level occupations may be low, especially the physical demands. However, the requirements of those occupations are often very specific, and the higher educated claimants are not able to return to their former higher level jobs because of health problems, and probably similar problems will occur if they try to work in other higher level jobs of a comparable nature. Moreover, they do not have the right specialisation to function in completely different occupations, and therefore they miss the opportunity to apply their specific work ability, as developed in their education and their former job [18]. To exaggerate: the long-term sick-listed employee, formerly working in a high-level job, is apparently not able to function in that job, and probably neither in similar jobs for which he/she is qualified, and for other high-level jobs he/she lacks (some of) the specific qualifications needed in those jobs. Therefore, it is probable that the (lower level) reference occupations will be the only opportunities for the high-educated claimants to work in a regular occupation. Hence, it is improbable that including more higher-level occupations in the job file would have resulted in the selection of a higher-level reference occupation. It is plausible that the composition of the sets of reference occupations, with only elementary and low level occupations, was caused by the additional barriers in the higher-level occupation for many claimants, leading to a low *a priori *maximum coverage. The reference occupations at the elementary and low level are useful to explore the general work ability [18].

The SCO-'92 occupational classification comprises 1,211 distinct occupational titles. Each occupational title covers jobs from a variety of companies and institutions, and therefore with a certain variation in specific working situations. A limited selection of these jobs within each occupation was included in the job file of the CAMS. This subgroup of jobs may not be a representative sample of all the jobs within the occupation in the Dutch work force, for example relatively low in the level of demands or required skills and education. However, each occupational title covers jobs from a variety of companies and institutions, and therefore with a certain variation in specific working situations. Not all of the jobs within the job file had been used by the LE in disability assessments of moderate severely disabled claimants. In these disability assessments, relatively demanding jobs had a lower probability of being used. Therefore, the choice of a reference occupation will probably be based on the use of a relatively accessible subgroup of jobs within the occupation in the job file. The description of the reference occupations, including the task demands, to be used in the newly proposed disability assessment procedure should be based on this accessible selection.

Our proposal for the set of reference occupations is based on the outcomes of real assessments of work disability in the public work disability insurance system in the Netherlands. The functional abilities of the claimants were used to look for dimensions within those abilities. In addition, the data of work disability assessments of severely disabled claimants within each dimension were used to identify jobs low in task demands in the corresponding dimension. This way of identification ensured the relevance of the reference occupations for the disabled employees involved. The large number of assessments makes the results stable, i.e. the probability is high that the same procedure would result in (almost) the same set of reference occupations, if it would be repeated in another cohort of claimants. However, it remains unknown whether these results can be generalised to other countries and/or social security systems. Nevertheless, the development of reference occupations might be very relevant for other countries, given the size and urgency of the problems in the work participation of disabled employees[1,3]. These reference occupations can be applied as an alternative method for the assessment of the work capacity of disabled employees in regular occupations as well as in rehabilitation efforts for those employees.

## Conclusion

In conclusion, the search for candidate occupations being eligible as reference occupations resulted in two proposals: 1) separate subsets for four subgroups of claimants, counting four to six occupations per set, totalling into a total set of 13 occupations (Table 4); and 2) a limited set of six occupations, applicable to all claimants (Table 5). The coverage of the former four subsets ranged from 84% to 93%, and was equal or higher than the coverage of the latter set: from 84% to 89%. The acceptance of the newly proposed method of disability assessment depends on the outcomes of the assessments in comparison with the outcomes of the present method. This requires a new research project, and the outcomes of that study will determine for an important part the choice to implement the newly proposed method. The choice between the two sets of reference occupations will also depend on a comparison of the outcomes. In case the outcomes/qualities of the two reference occupation sets are about equal, the latter set will probably be preferred, because of the smaller size of the total set and the use of the same set of reference occupations for all claimants. In such a research project, additional information can be gathered, for example: enquiries about the experiences of the professionals applying the new method; opinions about the pros and cons of the new method; etc. This additional information will help to improve the quality of the method and to promote the acceptance by the professionals. In the practice of occupational rehabilitation, the reference occupations can be used to assess the general working capacity[18], and to proceed from there, depending on the qualifications and motivation of the individual.

## Abbreviations

CAMS: Claim Assessment and Monitoring System; CBS: Statistics Netherlands CBS; ICF: International Classification of Functioning: Disability and Health; IP: Insurance physician; ISCO-88: International Standard Classification of Occupations; LE: Labour expert; LFA: List of Functional Abilities; NFAS: Norwegian Function Assessment Scale; OECD: Organisation for Economic Co-operation and Development; SCO-'92: Standard Classification on Occupations (the version of 1992); WHO: World Health Organisation.

## Competing interests

The authors declare that they have no competing interests.

## Authors' contributions

JPJB wrote the manuscript. HPGM, AJMS and AJvdB advised on the methods used, and commented on the manuscript. All authors have read and approved the final version of the manuscript.

## Pre-publication history

The pre-publication history for this paper can be accessed here:

http://www.biomedcentral.com/1471-2458/12/156/prepub
